# Circulating DNA in rheumatoid arthritis: pathological changes and association with clinically used serological markers

**DOI:** 10.1186/s13075-017-1295-z

**Published:** 2017-05-02

**Authors:** Elena Rykova, Aleksey Sizikov, Dirk Roggenbuck, Oksana Antonenko, Leonid Bryzgalov, Evgeniy Morozkin, Kseniya Skvortsova, Valentin Vlassov, Pavel Laktionov, Vladimir Kozlov

**Affiliations:** 1 Institute of Chemical Biology and Fundamental Medicine SB RAS, Novosibirsk, Russia; 2 Novosibirsk State Technical University, Novosibirsk, Russia; 3 Federal State Budgetary Scientific Institution “Research Institute of Fundamental and Clinical Immunology”, Novosibirsk, Russia; 4 Institute of Biotechnology, Brandenburg University of Technology Cottbus-Senftenberg, Senftenberg, Germany; 5 Institute of Molecular and Cellular Biology SB RAS, Novosibirsk, Russia; 6 Institute of Cytology and Genetics SB RAS, Novosibirsk, Russia; 7 Academician E. N. Meshalkin, Novosibirsk Research Institute of Circulation Pathology, Novosibirsk, Russia

**Keywords:** Rheumatoid arthritis, Circulating nuclear DNA, Mitochondrial DNA, Real-time PCR, Antibodies to citrullinated protein/peptide, Rheumatoid factor

## Abstract

**Background:**

Early diagnosis of rheumatoid arthritis (RA) is crucial to providing effective therapy and often hampered by unspecific clinical manifestations. Elevated levels of extracellular circulating DNA (cirDNA) in patients with autoimmune disease were found to be associated with etiopathogenesis. To our knowledge, this is the first study to investigate the putative diagnostic use of cirDNA in RA and its association with disease activity.

**Methods:**

Blood samples were taken from 63 healthy subjects (HS) and 74 patients with RA. cirDNA was extracted from plasma and cell surface-bound cirDNA fractions (csbDNA). cirDNA concentration was measured by quantitative real-time polymerase chain reaction. Rheumatoid factor was analyzed by immunonephelometry, whereas C-reactive protein and anticitrullinated protein/peptide antibodies (ACPA) were detected by enzyme-linked immunosorbent assay.

**Results:**

Plasma cirDNA was significantly elevated in patients with RA compared with HS (12.0 versus 8.4 ng/ml, *p* < 0.01). In contrast, nuclear csbDNA (n-csbDNA) was significantly decreased (24.0 versus 50.8 ng/ml, *p* < 0.01), whereas mitochondrial csbDNA (m-csbDNA) was elevated (1.44 × 10^6^ copies/ml versus 0.58 × 10^6^ copies/ml, *p* < 0.05) in RA. The combination of csbDNA (mitochondrial + nuclear) with ACPA reveals the best positive/negative likelihood ratios (LRs) for the discrimination RA from HS (LR+ 61.00, LR− 0.03) in contrast to ACPA (LR+ 9.00, LR− 0.19) or csbDNA (LR+ 8.00, LR− 0.18) alone.

**Conclusions:**

Nuclear and mitochondrial cirDNA levels in plasma and on the surface of blood cells are modulated in RA. Combination of cirDNA values with ACPA can improve the serological diagnosis of RA.

**Electronic supplementary material:**

The online version of this article (doi:10.1186/s13075-017-1295-z) contains supplementary material, which is available to authorized users.

## Background

The etiopathogenesis of rheumatoid arthritis (RA) is complex and involves both environmental and genetic factors. Early diagnosis of RA is of importance for effective therapy to prevent irreversible damage. However, RA diagnostics are hampered by the unspecific clinical manifestations [1]. One of the established clinically used RA markers is rheumatoid factor (RF), which represents a set of autoreactive antibodies against the immunoglobulin G (IgG) Fc fragment. According to authors of a recent meta-analysis, RF discriminates patients with RA from healthy subjects (HS) with only moderate sensitivity and specificity (69 and 85%, respectively) [2]. Moreover, elevated RF has been detected not only in patients with RA but also in patients with other systemic autoimmune rheumatic diseases. RF was the most significant diagnostic marker associated with RA until the discovery of anticitrullinated protein/peptide antibodies (ACPA). Initially, the presence of ACPA was discovered to be particularly useful diagnostically: It was more specific, although less sensitive, than the presence of RF [3]. Recent studies also suggested that ACPA positivity was predictive of RA months before the first clinical manifestations, and it was associated with more severe disease development and with better response to therapy with abatacept and adalimumab [4, 5].

The Disease Activity Score (DAS) is one of the most commonly used tools to monitor disease activity in RA. C-reactive protein (CRP) is a sensitive but unspecific inflammation marker that is widely used for the 28-joint Disease Activity Score (DAS28) based on C-reactive protein definition in patients with RA and is in good agreement with DAS28 based on erythrocyte sedimentation rate [6].

Increased cell-free nuclear circulating DNA (n-cirDNA) was detected in the blood plasma/sera of patients with certain disorders, such as cancer and autoimmune diseases, in particular patients with systemic lupus erythematosus (SLE) [7–10]. Mitochondrial circulating DNA (m-cirDNA) was also elevated in patients with cancer and disorders associated with massive cell damage, such as acute ischemic stroke [11], myocardial infarction [12], trauma [13], and severe sepsis [14]. The biological role of n/m-cirDNA remains enigmatic in both health and pathology. Recently, attention has been focused on the characterization of a possible alternative form of cell-free nuclear and mitochondrial DNA acting as an autoantigen in triggering SLE [15]. Accumulating data indicate that an increased amount of n/m-cirDNA in plasma can be associated with autoimmune pathogenesis, especially in SLE and Sjögren’s syndrome [7, 9, 16, 17]. One research group used parallel genomic and methylomic sequencing for the comparison of plasma cell-free circulating DNA (cirDNA) and discovered a number of plasma DNA abnormalities in patients with SLE in contrast to HS [18].

n-cirDNA was also found on the surface of blood cells—both erythrocytes and leukocytes. In patients with cancer, cell surface-bound DNA (csbDNA) demonstrated changes of concentration and composition due to accumulation of DNA molecules coming from cancer cells [19, 20]. Our observations indicate a continuous exchange between circulating cell-bound and cell-free nuclear DNA (nDNA) pools in whole blood [20]. Thus, the goal of this study was to investigate the occurrence of n/m-cirDNA in patients with RA and its association with disease activity. Accordingly, levels of n/m-cirDNA and n/m-csbDNA were determined in patients with RA to assess their association with RA development and to evaluate their potential as RA markers in combination with the routinely used RF, CRP, and ACPA.

## Methods

### Patients

Blood samples were taken from 63 HS and 74 randomly selected patients with RA who were under care at the Affiliated Clinics of the Research Institute of Fundamental and Clinical Immunology (Novosibirsk, Russia) and fulfilled the 1987 American College of Rheumatology criteria for RA (group 1) (Table 1) [21]. Patient clinical data, including sex, age, disease activity, and number of affected joints, were obtained from the clinic’s registry (see Table 1). The assessments of RA disease activity were performed with the DAS28 rating system, which comprised the following ratings: <3.2 for low activity (class I), 3.2–5.1 for moderate activity (class II), and >5.1 for high activity (class III). Patients with RA recruited for the study received methotrexate (MTX) (20 mg/week) and etoricoxib (90 mg/day). Therapy was started at least 3 months before blood sampling. In addition, folic acid (5 mg/week 1 day after MTX treatment) was administered to all patients.

**Table 1:** Characteristics of patients and healthy subjects

	RA subgroup 1	RA subgroup 2	HS
Number of subjects	74	14	63
Sex, female, *n* (%)	65 (88%)	12 (85%)	52 (82%)
Age, years, median (range)	54.7 (16–76)	49.3 (34–63)	51.2 (23–74)
DAS28, median (range)	5.5 (3.5–7.02)	3.2 (2.7–3.5)	–
Number of subjects with classes I–II DAS28/class III DAS28	28/46	14/0	–
Number of subjects with recent-onset/established/end-stage RA	5/44/25	0/14/0	–

*DAS28* 28-joint Disease Activity Score, *RA* Rheumatoid arthritis, *HS* Healthy subjects

*See* Methods for descriptions of RA subgroups 1 and 2

Another group of patients with RA (group 2) (Table 1) included 14 patients with active disease who did not respond to MTX and were treated with one (*n* = 11) or two courses (baseline and month 6) (*n* = 3) of rituximab plus MTX. Rituximab (1000 mg) was administered intravenously on day 1 and day 15. All patients enrolled in the study achieved a European League Against Rheumatism (EULAR) moderate/good response at week 24. Pathologic conditions known to be associated with high cell-free DNA release, including neoplastic and infectious diseases, surgery or major trauma, hemodialysis, and pregnancy, were not observed in patients and control subjects.

### Preparation of plasma and cell surface-bound fractions and DNA extraction

Venous blood samples were stabilized and fractionated into plasma and blood cells by centrifugation. Cell surface-bound fractions containing n-csbDNA and m-csbDNA were obtained as previously described [22]. Briefly, blood cells were incubated with 9 vol of PBS containing 5 mM ethylenediaminetetraacetic acid (PBS-EDTA) for 5 minutes at room temperature. The cells were pelleted by centrifugation for 20 minutes at 800 × *g* and incubated with an equal volume of 0.25% trypsin solution. The enzyme was then inactivated using a trypsin inhibitor derived from soybeans (Sigma-Aldrich, St. Louis, MO, USA) for 4 minutes at room temperature. Plasma, PBS-EDTA, and trypsin eluates were centrifuged for an additional 20 minutes at 2000 × *g*, and aliquots were stored frozen at −80 °C. Purified cirDNA samples were obtained using a blood DNA isolation kit (BioSilica Ltd., Novosibirsk, Russia).

### Measurement of nuclear DNA and mitochondrial DNA concentrations in the blood

n-cirDNA concentration was measured by quantitative polymerase chain reaction (PCR) specific for long interspersed nuclear element 1 (LINE-1) repetitive elements as described elsewhere [23]. The real-time PCR of LINE-1 sequences was performed using an iCycler thermal cycler (Bio-Rad Laboratories, Hercules, CA, USA) in a total reaction volume of 30 μl containing 5 μl of extracted DNA; 600 nM of each primer (forward 5′-TTC AAC AAG AAG AGC TAA CTA TCC-3′, reverse 5′-TTG TAG GTC ACT CAG GAC TTG C-3′); 300 nM probe (5′-[5, 6]carboxytetramethylrhodamine [5,6-TAMRA]-TGC ACC CAA TAC AGG AGC ACC CAG ATT CA-black hole quencher 2 (BHQ2)-3′); 2.5 mM deoxynucleotide triphosphates (dNTPs); and Taq polymerase buffer containing 6 mM MgCl_2_ and 1 U of Taq polymerase, which was kindly provided by the Laboratory of Immunogenetics of the Institute of Molecular and Cellular Biology, Russian Academy of Sciences, Siberian Branch. PCR was performed under the following conditions: denaturation at 95 °C for 4.5 minutes, followed by 45 cycles at 95 °C for 15 seconds and 60 °C for 45 seconds. Standard curves were generated using serial dilutions of the purified and quantified nDNA from human leukocytes.

m-cirDNA concentration was evaluated by quantitative PCR specific for a 172-bp mitochondrial DNA segment between nucleotide positions 3130 and 3301 [GenBank accession number J01415] [24]. The real-time PCR was performed using an iCycler thermal cycler in a total reaction volume of 30 μl containing 5 μl of extracted DNA, 600 nM of each primer (forward 5′-AGG-ACA-AGA-GAA-ATA-AGG-CC-3′, reverse 5′-TAA-GAA-GAG-GAA-TTG-AAC-CTC-TGA-CTG-TAA-3′), 300 nM probe (5′-[5, 6]-TAMRA-TTC-ACA-AAG-CGC-CTT-CCC-CCG-TAA-ATG-A-BHQ2-3′), 2.5 mM dNTPs, and Taq polymerase buffer containing 6 mM MgCl_2_ and 1 U of Taq polymerase. PCR was performed under the following conditions: denaturation at 95 °C for 4.5 minutes, followed by 45 cycles of 95 °C for 15 seconds and 56 °C for 45 seconds.

### Measurement of CRP and autoantibodies

CRP was determined by using an immunonephelometric assay kit (Olvex Diagnosticum, St. Petersburg, Russia). RF in blood plasma was detected by using a commercially available enzyme-linked immunosorbent assay (ELISA) (Vector-Best, Novosibirsk, Russia). ACPA was tested as anticyclic citrullinated peptide 2 antibody (anti-CCP2). Baseline anti-CCP2 antibody status (positive/negative) and concentration were determined using an anti-CCP2 IgG ELISA kit (Medizym anti-CCP Ref; Medipan, Berlin, Germany). Patients with a baseline anti-CCP2 IgG concentration ≥25 U/ml were considered to be positive according to the manufacturer’s specifications.

### Statistical analysis

The Mann-Whitney *U* test and analysis of variance (ANOVA) package in R were used to assess the statistical relationships between the analyzed factors and the clinical variables. A correlation analysis was conducted with Spearman’s rank correlation test. Principal component analysis (PCA) allowed visualizing representation of point class factorial maps. Random forest classification algorithm developed by Breiman and implemented as the randomForest package in R was used to estimate the performance of a predictive model [25, 26]. randomForest is an ensemble classifier in which the base classifier is an unpruned decision tree built from a random selection of feature variables for a randomly selected subset of training samples (patients). The method enables evaluation of the effect of a feature variable upon the classification, identified as the importance score. Using the randomForest package (v.4.6-2) in the R programming language, a random forest of 10,000 trees was generated for classification.

## Results

### Circulating nuclear DNA, mitochondrial DNA, ACPA, RF, and CRP concentrations in healthy subjects and patients with RA

A significant increase of the plasma n-cirDNA concentration was found for patients with RA compared with age- and sex-matched HS (see Table 1) (median 12.0 versus 8.4 ng/ml, *p* < 0.05 by Mann-Whitney *U* test), whereas levels of n-csbDNA in patients with RA were found to be significantly decreased (24.0 versus 50.8 ng/ml, *p* < 0.01) (Table 2). m-cirDNA concentration was not changed in patients with RA compared with HS (median 0.38 × 10^6^ copies/ml versus 0.34 × 10^6^ copies/ml, *p* > 0.05), whereas the m-csbDNA level was different (1.44 × 10^6^ copies/ml versus 0.58 × 10^6^ copies/ml, *p* < 0.05). As expected, significant elevations of RF (median 16.1 versus 6.8 U/ml, *p* < 0.01), CRP (median 21.4 versus 2.6 U/ml, *p* < 0.01), and ACPA (median 1725 versus 15 U/ml, *p* < 0.01) were detected in patients with RA compared with HS (Table 2). ANOVA of cell-free n-cirDNA and cell-free m-cirDNA circulating in the blood plasma did not reveal significant discriminative power between patients with RA and the control groups (Table 3). According to ANOVA, the most valid parameters for the discrimination of patients with RA from control subjects were ACPA, CRP, m-csbDNA, and RF levels (*F* = 48.4, *Pr* < 0.001, *F* = 30.3, *Pr* < 0.001; *F* = 17.7, *Pr* < 0.001, and *F* = 16.3, *Pr* < 0.001, respectively), whereas n-csbDNA, n-cirDNA, and m-cirDNA possessed lower power for discrimination of patients and control subjects (Table 3).

**Table 2:** Median concentrations of nuclear and mitochondrial circulating DNA and cell surface-bound DNA as well as C-reactive protein and autoantibodies in blood from patients with rheumatoid arthritis and healthy subjects

	n-cirDNA^a^	n-csbDNA^a^	m-cirDNA^b^	m-csbDNA^b^	RF^c^ (U/ml)	ACPA^c^ (U/ml)	CRP^c^ (mg/L)
HS	8.4 (1.6–115.2)	50.8 (4–368.4)	0.34 × 10^6^ (0.06–1.34) × 10^6^	0.58 × 10^6^ (0.14–0.24) × 10^6^	6.8 (2.6–13.9)	15 (2–32)	2.6 (0.9–8.1)
Patients with RA^d^	12.0 (0.4–183.2)	24.0 (1.8–290.8)	0.38 × 10^6^ (0.006–3.38) × 10^6^	1.44 × 10^6^ (0.06–9.36) × 10^6^	16.1 (0.4–169)	1725 (4–3254)	21.4 (0.4–169)
HS versus RA	*p* < 0.05	*p* < 0.01	*p* > 0.05	*p* < 0.01	*p* < 0.01	*p* < 0.01	*p* < 0.01
RA activity classes I–II (subgroup 1)	10.4 (2.8–32.8)	21.6 (1.8– 160.0)	0.32 × 10^6^ (0.08–1.68) × 10^6^	0.96 × 10^6^ (0.068–5.18) × 10^6^	10 (4.1–130)	1370 (4 – 2942)	11.9 (1.4–139)
RA activity class III (subgroup 2)	14.0 (0.4–183.2)	25.6 (2.8–290.8)	0.42 × 10^6^ (0.06–3.4) × 10^6^	1.54 × 10^6^ (0.26–9.36) × 10^6^	16.3 (0.4–169)	1945 (10 – 3254)	27.6 (1.6–147.1)
Subgroup 1 versus subgroup 2	*p* < 0.05	*p* > 0.05	*p* > 0.05	*p* > 0.05	*p* > 0.05	*p* > 0.05	*p* < 0.05

*Abbreviations: ACPA* Anticitrullinated protein/peptide antibodies, *CRP* C-reactive protein, *HS* Healthy subjects, *m-cirDNA* Cell-free mitochondrial circulating DNA, *m-csbDNA* Mitochondrial cell surface-bound DNA, *n-cirDNA* Cell-free nuclear circulating DNA, *n-csbDNA* Nuclear cell surface-bound DNA, *RA* Rheumatoid arthritis, *RF* Rheumatoid factor

^a^n-cirDNA and n-csbDNA concentrations in nanograms per milliliter of blood

^b^m-cirDNA and m-csbDNA concentrations in copies per milliliter of blood

^c^RF, ACPA, and CRP concentrations in units per milliliter of blood plasma; median values are presented with range in parentheses

^d^RA group 1 (*n* = 74) (*see* Table 1)

**Table 3:** Contributions of different variables to intergroup differences of patients with rheumatoid arthritis versus healthy subjects based on analysis of variance

Variable	RA^a^ versus control		Activity^b^	
	*F*	*Pr* (>*F*)	*F*	*Pr* (>*F*)
ACPA	48.4	<0.001	1.46	0.23
n-cirDNA	3.73	0.06	1.67	0.20
n-csbDNA	6.07	0.02	0.28	0.60
m-cirDNA	3.95	0.05	0.35	0.55
m-csbDNA	17.7	0.00005	2.59	0.11
C-reactive protein	30.3	2.64e-07	1.27	0.26
Rheumatoid factor	16.3	0.0001	1.81	0.18

*Abbreviations: ACPA* Anticitrullinated protein/peptide antibodies, *m-cirDNA* Cell-free mitochondrial circulating DNA, *m-csbDNA* Mitochondrial cell surface-bound DNA, *n-cirDNA* Cell-free nuclear circulating DNA, *n-csbDNA* Nuclear cell surface-bound DNA, *RA* Rheumatoid arthritis

^a^ RA group 1 (*n* = 74) (see Table 1)

^b^ Patients with RA were divided into two groups according to RA activity score: group 1 with low and moderate activity (classes I–II DAS28) and group 2 with high activity of the disease (class III DAS28)

### Correlation of circulating nuclear DNA, mitochondrial DNA, ACPA, RF, and CRP with demographic characteristics

In terms of sex distribution, 88% of patients with RA and 82% of the control group recruited into the study were female (see Table 1). We did not find significant sex-associated differences for cell-free n-cirDNA, m-cirDNA in blood plasma, n-csbDNA, m-csbDNA, ACPA, RF, or CRP in both groups (*p* > 0.05). According to the Spearman rank-order correlation test, cell-free n-cirDNA, m-cirDNA in blood plasma, csb-cirDNA, m-csbDNA, CRP, and RF levels did not show a significant correlation with age in patients with RA (*p* > 0.05). However, there was a moderate positive association of ACPA with age (*r* = 0.39, *p* = 0.003) (Additional file 1: Figure S1).

In healthy donors, a weak correlation of plasma n-csbDNA and m-csbDNA did not reach significance (*r* = 0.24, *p* = 0.06) (Additional file 2: Figure S2). In patients with RA, cell-free plasma n-cirDNA showed a moderate positive correlation with CRP (*r* = 0.42, *p* < 0.001) (Additional file 1: Figure S1). Furthermore, ACPA demonstrated a weak positive correlation with RF (*r* = 0.28, *p* = 0.02) and a weak negative correlation with m-cirDNA (*r* = −0.27, *p* = 0.02).

### Association of circulating nuclear DNA, mitochondrial DNA, ACPA, RF, and CRP levels with disease activity and treatment

All patients fulfilled the criteria for DAS28 classes I, II, and III. According to Spearman’s test, a positive correlation was found between DAS28 and CRP levels (*r* = 0.38, *p* = 0.019). n-cirDNA levels demonstrated a tendency for a significant correlation with disease activity based on DAS28 (*r* = 0.31, *p* = 0.06).

**Fig. 1:**
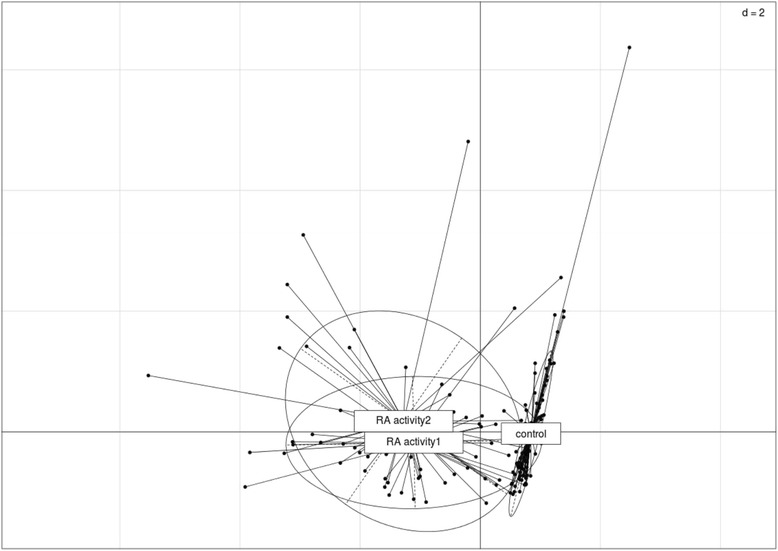
Challenge data inspection by principal component analysis. Scatterplots with representation of the various classes were produced with the s.class command of the ade4 R package. The various classes are control healthy donors, rheumatoid arthritis (RA) activity 1 (with classes I–II RA activity), RA activity 2 (with class III RA activity). The variables analyzed are: cell surface-bound nuclear DNA, cell surface-bound mitochondrial DNA, anticitrullinated protein/peptide antibodies, and rheumatoid factor

Patients were divided into two subgroups according to disease activity score: subgroup 1 (with low and moderate activity in classes I–II DAS28) and subgroup 2 (with high activity of the disease in class III DAS28). Median n-cirDNA and CRP plasma levels were significantly higher in RA subgroup 2 with higher disease activity than in RA subgroup 1 and control subjects according to the Mann-Whitney *U* test (*p* < 0.05) (Table 2). None of the other five variables (n-csbDNA, m-csbDNA, ACPA, and RF) estimated in the study showed discriminative power between these two subgroups according to Mann-Whitney *U* test and ANOVA (Tables 2 and 3). The use of PCA (Monte Carlo test) demonstrated an association of RA disease with four selected variables: n-csbDNA, m-csbDNA, ACPA, and RF. In contrast, no significant association with disease activity was revealed (Fig. 1). Representation of point class factorial maps allowed visualizing discrimination of two subgroups of patients with RA (subgroups 1 and 2) from the control group.

Patients with RA were further divided into three subgroups according to their RA clinical classification (recent onset, established, and end stage) [27]. According to Mann-Whitney *U* test results, n-cirDNA, m-cirDNA, n-csbDNA, and m-csbDNA levels did not differ between these subgroups (*p* > 0.05) (Additional file 3: Table S1). Median plasma CRP levels showed steady increases in the following direction: recent-onset RA < established RA < end-stage RA (11.3, 19.1, and 27.4 mg/L, respectively) (recent-onset versus end-stage RA, *p* < 0.05). Plasma RF level was significantly higher in patients with RA with established and end-stage RA than in patients with recent-onset RA (23.1 and 16.1 versus 4.0 mg/L, respectively; *p* < 0.01 for both comparisons) (Additional file 3: Table S1). The prevalence of ACPA-negative results was higher in patients with recent-onset RA than in those with established and end-stage RA (33%, 17%, and 9%, respectively), but the differences did not reach significance.

One inclusion criterion for the first group of patients with RA (*n* = 74) in our study was a uniform therapeutic treatment with MTX and etoricoxib along with folic acid cotherapy, which, according to earlier reports, should not influence disease-associated circulating DNA changes (see Discussion). According to the Spearman’s rank-order correlation test, cell-free n-cirDNA in blood plasma (*r* = 0.01, *p* = 0.97), cell-free m-cirDNA in blood plasma (*r* = 0.024, *p* = 0.86), n-csbDNA (*r* = 0.08, *p* = 0.54), and m-csbDNA (*r* = −0.048, *p* = 0.73) did not show a significant correlation with therapy duration. To evaluate the association of circulating nDNA and mitochondrial DNA with patient response to different treatment, we studied an additional group of patients (*n* = 14) treated with the biologic disease-modifying antirheumatic drug rituximab with MTX, and we found that they achieved a EULAR moderate/good response at week 24 (*see* Table 1). According to Mann-Whitney *U* test results, n-cirDNA, m-cirDNA, n-csbDNA, and m-csbDNA levels did not differ between these two groups (*p* > 0.05) (Additional file 4: Table S2). Notably, m-csbDNA levels in patients treated with rituximab showed a tendency to be lower than in patients treated with MTX and etoricoxib (0.93 × 10^6^ versus 1.44 × 10^6^), although the difference did not reach significance (*p* = 0.15).

### Circulating nuclear DNA, mitochondrial DNA, ACPA, RF, and CRP combinations as potential RA diagnostic markers

**Fig. 2:**
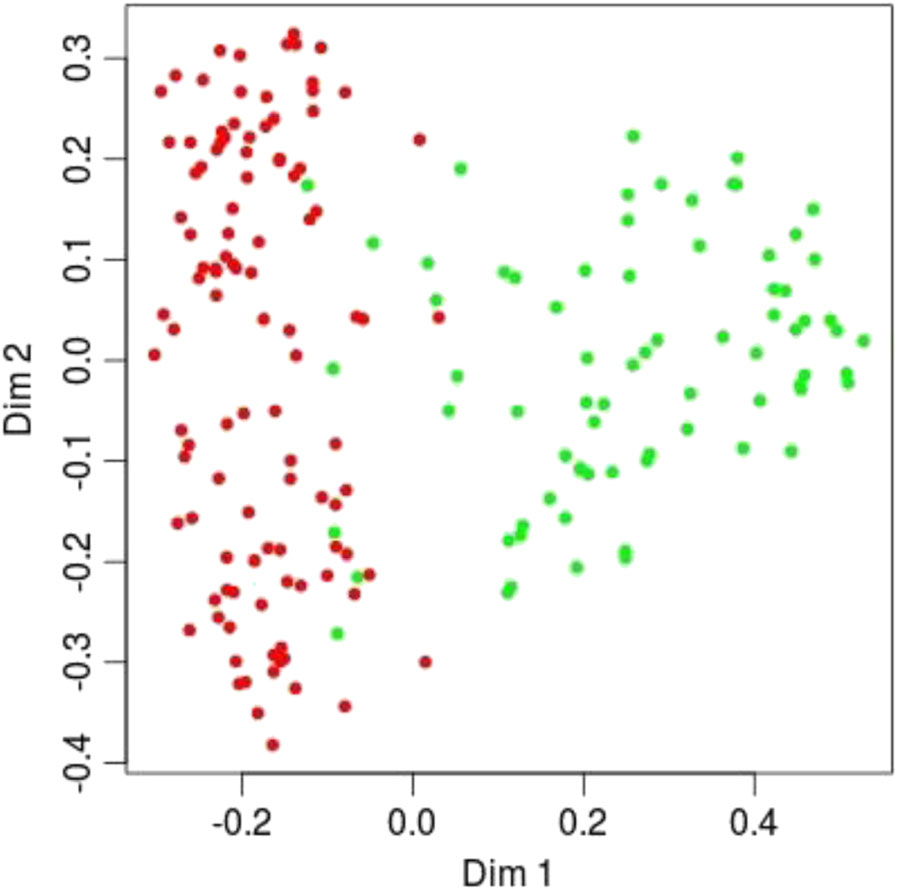
Random forest classification tree plot. Diagram with visualization of patients with rheumatoid arthritis and discrimination from healthy donors by random forest classification tree algorithm, based on cell surface-bound mitochondrial DNA and anticitrullinated protein/peptide antibodies estimates. *Red circles* represent individual healthy donors; *green circles* represent individual patients with rheumatoid arthritis

We selected five factors that were significantly modulated in patients with RA compared with HS according to Mann-Whitney *U* test results (Table 2) and that demonstrated the highest power for discrimination of patients with RA according to ANOVA (Table 3). We further tested the predictive accuracy for RA diagnostics on the basis of combination of n-csbDNA and m-csbDNA levels, as well as their combination with ACPA, RF, and CRP plasma levels. Using the machine learning random forests test with two variables (n-csbDNA + M-csbDNA), we could discriminate patients with RA from HS with 84% sensitivity and 89% specificity (Table 4). The combination of the routinely used markers RF and CRP revealed 86% sensitivity and 84% specificity. ACPA alone demonstrated 83% sensitivity and 90% specificity, whereas the combination of ACPA + RF + CRP improved the diagnostic power (90% sensitivity and 94% specificity). Notably, combination of ACPA with two circulating DNA markers (ACPA + n-csbDNA + m-csbDNA) also provided high accuracy for discrimination of patients with RA from HS (97% sensitivity and 98% specificity) (Table 4). Moreover, the two-marker-based panel (ACPA + m-csbDNA) allowed discrimination of patients with RA from HS with 91% sensitivity and 98% specificity. Figure 2 depicts the separate clustering of HS from patients with RA, whereas patients with RA from subgroups 1 and 2 demonstrate joint clustering; this analysis is based on ACPA + m-csbDNA combination. The importance of quantitative components evaluated using the random forest algorithm decreased in the following sequence: ACPA > m-csbDNA > CRP > RF > n-csbDNA (Additional file 5: Table S3).

**Table 4:** Evaluation of a diagnostic test of marker combinations in healthy donors versus patients with rheumatoid arthritis^a^

Markers	Sensitivity	Specificity	LR+ (95% CI)	LR− (95% CI)
n-csbDNA + m-csbDNA	84% (73.26% to 91.76%)	89% (78.44% to 95.41%)	8 (3.73 to 15.33)	0.18 (0.10 to 0.31)
ACPA	83% (71.59% to 90.68%)	90% (80.41% to 96.42%)	9 (4.02 to 18.71)	0.19 (0.11 to 0.32)
RF + CRP	86% (74.96% to 92.83%)	84% (72.74% to 92.12%)	5 (3.03 to 9.59)	0.17 (0.10 to 0.31)
ACPA + RF + CRP	90% (80.21% to 95.82%)	94% (84.53% to 98.24%)	14 (5.46 to 36.66)	0.11 (0.05 to 0.22)
ACPA + m-csbDNA	91% (82.03% to 96.74%)	98% (91.47% to 99.96%)	57 (8.22 to 402.59)	0.09 (0.04 to 0.19)
ACPA + m-csbDNA + RF	93% (83.89% to 97.61%)	97% (89.00% to 99.61%)	29 (7.46 to 114.45)	0.07 (0.03 to 0.17)
ACPA + n-csbDNA + m-csbDNA	97% (89.92% to 99.65%)	98% (91.47% to 99.96%)	61 (8.75 to 427.75)	0.03 (0.01 to 0.12)

*Abbreviations*: *LR+* Positive likelihood ratio, *LR*− Negative likelihood ratio, *ACPA* Anticitrullinated protein/peptide antibodies, *CRP* C-reactive protein, *m-cirDNA* Cell-free mitochondrial circulating DNA, *m-csbDNA* Mitochondrial cell surface-bound DNA, *n-cirDNA* Cell-free nuclear circulating DNA, *n-csbDNA* Nuclear cell surface-bound DNA, *RF* Rheumatoid factor

^a^Rheumatoid arthritis group 1 (*n* = 74) (see Table 1)

The positive and negative likelihood ratios (LR+ and LR−, respectively) were calculated for combinations of five selected RA markers (Table 4). LR+ values increase in the following sequence: ACPA < ACPA + RF + CRP < ACPA + m-csbDNA < ACPA + n-csbDNA + m-csbDNA (9, 14, 57, and 61, respectively). LR− showed a value <0.1 in cases of two combinations (ACPA + m-csbDNA and ACPA + n-csbDNA + m-csbDNA), which indicates that these combinations make significant changes in the posttest probabilities in case of the negative result (Table 4).

## Discussion

To our knowledge, this is the first study to simultaneously quantify circulating nDNA and mitochondrial DNA in blood plasma, as well as nDNA and mtDNA bound on the surface of blood cells of patients with RA. The study demonstrates significant differences of circulating cell surface-bound nDNA and cell surface-bound mitochondrial DNA levels in patients with RA compared with HS. Nuclear and mitochondrial DNA fragments were found at low levels in plasma/serum from HS and were elevated in patients with cancer and certain disorders associated with the increase of cell damage or death (autoimmune disorders, trauma, stroke) [7–14]. The associated changes of mitochondrial DNA and nDNA in the blood suggest identical sources of their origin, such as probably cell death remnants along with complexes and vesicles secreted by living cells [19, 28–31].

Furthermore, circulating plasma/serum nDNA levels were reported to be elevated in patients with different systemic autoimmune disorders. In a recent study, Bartoloni et al. detected significant changes of n-cirDNA levels in 48 patients with SLE and 44 patients with Sjögren’s syndrome compared with control subjects [10]. Also, Chen et al. [9] demonstrated n-cirDNA increases in SLE; however, two earlier reports showed no changes in SLE compared with healthy individuals [7, 32]. Patients with systemic sclerosis developed increased n-cirDNA levels only in the case of active disease [8]. These controversial data could be the result of different protocols of blood sample processing and n-cirDNA extraction and analysis.

In our study, the plasma n-cirDNA level in patients with RA was increased, which is in accordance with two earlier reports [33, 34]. In contrast, the level of n-csbDNA was decreased in patients with RA compared with HS. Earlier, we detected decreased levels of n-csbDNA in patients with breast, lung, and prostate cancer, which was not observed in patients with benign tumors [19, 22]. Multiple reasons can be responsible for the decreased binding of n-cirDNA to cell surfaces in patients with RA, including the characteristics of circulating DNA-protein complex modification as well as disease-induced changes in the composition and amount of the blood cell surface proteins and plasma proteins. Recently, a link between circulating cell-free DNA levels and neutrophil extracellular trap (NET) formation was established in a number of autoimmune conditions, including RA [35]. NETs are extruded by polymorphonuclear neutrophils (PMNs) and consist of chromosomal DNA complexed with antimicrobial peptides and proteases. Earlier studies suggested that RA-derived PMNs were more prone to undergo NETosis and that components of NETs, including circulating DNA, could contribute to the generation of autoantigens [35].

Recent reports showed increased plasma levels of total microparticles (MPs) in patients with as RA, SLE, and Sjögren’s syndrome compared with healthy donors [36, 37]. MPs are known to contain fragmented nDNA, and thus the increase of plasma n-cirDNA levels may be a result of systemic MP release due to constant cytokine stimulation in steroidal anti-inflammatory drugs [38]. Nielson et al. evaluated the putative role of MPs in SLE as circulating antigenic targets and carriers of autoimmune complexes [37]. Zhong et al., using protein G sepharose bead adsorption of plasma to isolate antibody-bound DNA [34], reported an association of elevated n-cirDNA with higher DNA-binding antibody levels in the plasma of patients with RA. They concluded that the content/composition of DNA-bearing complexes in the circulation of patients with RA differed from that in a control group [34].

This study demonstrates an increase of cell surface-bound mitochondrial DNA in patients with RA compared with HS, whereas plasma m-cirDNA in patients with RA was not modulated. Researchers in several recent studies reported elevated levels of plasma m-cirDNA, too, and discussed their diagnostic significance in breast, prostate, bladder, renal cell, and testicular germ cell cancers [39–42]. Significantly elevated levels of plasma m-cirDNA have been found in patients with other clinical conditions associated with massive cell damage, such as acute ischemic stroke [11], trauma [13], and severe sepsis [14]. Interestingly, extracellular mitochondrial DNA was shown to be recognized by immune cells as a danger signal and to trigger an inflammatory response, such as in cases of posttraumatic shock [43]. Mitochondrial DNA possess immunomodulatory properties of bacterial DNA as a result of mitochondrial endosymbiotic origin. Probably owing to its proinflammatory potential, m-cirDNA in plasma was recently demonstrated as a potent predictor of posttraumatic systemic inflammatory response syndrome, multiple organ dysfunction syndrome [44, 45], and intensive care unit patient mortality [46]. Despite the accumulating evidence of cell-free mitochondrial DNA-induced immune deregulation, there is only one study on m-cirDNA in autoimmune disorders. Hajizadeh et al. described increased m-cirDNA levels in plasma and synovial fluid of patients with RA [47]. Those authors proposed the involvement of m-cirDNA in joint inflammation by activating immune cells to produce proinflammatory cytokines. However, the mechanisms leading to autoreactivity induction by mitochondrial DNA remain elusive. Mitochondrial DNA released into the extracellular milieu and circulation under certain conditions has potential to trigger autoreactivity because of being totally CpG-unmethylated [48, 49]. A recent study on DNase II-knockout mice demonstrated that the mice developed symmetrical erosive polyarthritis. This indicates that cell-free nDNA mitochondrial DNA itself can be directly involved in the etiology of RA [50, 51]. In these mice, the ability of macrophages to degrade DNA from erythrocyte precursors and apoptotic cells was severely reduced, and immune cells produced large quantities of proinflammatory cytokines, RF, and antinuclear antibodies.

Today, the most informative RA-specific serological marker is the occurrence of autoantibodies to citrullinated proteins. According to recent reports, the autoimmune response against citrullinated proteins develops in correlation with autoreactive antibodies against the self IgG Fc fragment (RF). The combination of these two markers enables higher accuracy of RA diagnostics [52]. In accordance with earlier observations [53], the present study demonstrated a positive correlation of ACPA with RF levels in patients with RA. In contrast, we found a negative correlation of ACPA with m-csbDNA and n-csbDNA levels. These data suggest that ACPA/RF, on one hand, and m-csbDNA, n-csbDNA, on the other hand, could be independent circulating markers of RA development and that their combination may provide a powerful diagnostic tool.

According to our results, the combination of two circulating markers (m-csbDNA + n-csbDNA) had a sensitivity of 84% and specificity of 89% for RA diagnosis in patients with different disease activity levels and stages. The combination of the routinely used ACPA, RF, and CRP resulted in 90% sensitivity and 94% specificity. Addition of the two circulating DNA markers to ACPA (ACPA + m-csbDNA + n-csbDNA) showed the highest power for the discrimination of RA from HS (97% sensitivity and 98% specificity). This resulted in an improved LR− of the blood-based test (0.19 versus 0.03).

Our study, in accord with earlier reports, indicates that RF and CRP levels are dependent on disease stage and activity. In contrast, ACPA, m-csbDNA, and n-csbDNA changes are found not only in end-stage RA but also in recent-onset/established RA stages and are not associated with disease activity; therefore, these parameters look promising as diagnostic markers. One inclusion criterion for patients with RA in our study was a uniform therapeutic treatment with MTX and etoricoxib along with folic acid cotherapy. Etoricoxib belongs to the class of nonsteroidal anti-inflammatory drugs acting as cyclooxygenase 2 inhibitors with no reported influence on DNA synthesis [54]. MTX is a disease-modifying antirheumatic drug that produces measurable cytotoxic effects in cancer therapy when administered in short courses at very high doses (up to 1000 mg). The induced depletion of folate-dependent thymidine and purine residues, and thus antagonism of DNA synthesis as well as cell-cycle arrest at S_1_, are well established. However, these are not the same mechanisms by which low-dose MTX exerts its therapeutic effect in RA [55]. Evidence derived from clinical practice does not support the purine-pyrimidine antagonism hypothesis in RA treatment. The concomitant administration of folate once per week is believed to reduce the incidence of treatment-related adverse effects as well as liver function abnormalities and does not result in a loss of clinical benefit [56]. Smolenska et al. reported that a single-dose MTX treatment in patients with RA reduced the concentration of uric acid and hypoxanthine in whole blood for 1 day and then returned to the pretreatment levels within 2 days without any concomitant change in blood adenosine levels [57]. However, the influence of MTX in combination with folic acid on whole-blood purine levels was not studied. The present study reveals circulating DNA changes associated with RA development in patients who received a uniform treatment. To our knowledge, this treatment should not influence disease-associated circulating DNA changes. The folate cotherapy should abrogate MTX’s supposed effect on folate-dependent purine-pyrimidine synthesis and its putative influence on cirDNA levels. Spearman’s rank-order correlation test demonstrated no correlation of circulating DNA levels with the duration of therapy ranging from 3 months to 5 years in our study.

To clarify the question whether circulating DNA level is changed in patients in response to a different therapy, we studied the group of patients with active disease who did not respond adequately to MTX and therefore were treated with the B-cell-depleting biological agent rituximab. It is of interest that m-csbDNA levels in this group of patients showed a tendency to be decreased compared with the group of MTX/etoricoxib-treated patients. One can propose this change to be associated with the positive response to rituximab; however, this effect should be validated in a larger group of patients. Further studies evaluating cirDNA as a biomarker of RA therapy efficiency in differentially treated patients can provide information on therapeutic effects on cirDNA in RA.

A limitation of our study is the low number of patients with early RA as well as that a group of high-risk persons should have been examined. Future investigations are warranted to assess the value of m-csbDNA and n-csbDNA for an early noninvasive diagnostic test in combination with ACPA. An additional point that needs further evaluation is the specificity of the combination of m-csbDNA, n-csbDNA, and ACPA regarding the discrimination of RA from other autoimmune diseases.

## Conclusions

The development of RA is accompanied by significant changes of circulating nuclear and mitochondrial DNA levels found both in plasma and at the surface of blood cells. Our data provide evidence that quantitative analysis of n-csbDNA, m-csbDNA, and especially their combination with ACPA levels is a useful tool for the diagnosis of patients with RA with different disease stages and activity levels. The clinical usefulness of the suggested marker set derived from this study remains to be validated in larger cohorts of patients with early and late RA.

## Additional files

Additional file 1: Figure S1. Correlation of n-cirDNA, m-cirDNA, n-csbDNA, m-csbDNA, ACPA, CRP, and RF between each other and with age of patients with rheumatoid arthritis. (DOC 220 kb)

Additional file 2: Figure S2. Correlation of n-cirDNA, m-cirDNA, n-csbDNA, m-csbDNA, ACPA, CRP, and RF between each other and with age of healthy subjects. (DOC 219 kb)

Additional file 3: Table S1. Comparison of cir-nDNA, m-cirDNA, n-csbDNA, and m-csbDNA concentration in the blood from patients with recent-onset, established, and end-stage rheumatoid arthritis. (DOC 31 kb)

Additional file 4: Table S2. cir-nDNA, m-cirDNA, n-csbDNA, and m-csbDNA concentration in the blood from patients treated with methotrexate plus etoricoxib in comparison with rituximab plus methotrexate. (DOC 49 kb)

Additional file 5: Table S3. Variable importance evaluated using random forests classification algorithm. (DOC 28 kb)

## Abbreviations

ACPA: Anticitrullinated protein/peptide antibodies
ANOVA: Analysis of variance
anti-CCP2: Anticyclic citrullinated peptide 2 antibody
BHQ2: Black hole quencher 2
cirDNA: Cell-free circulating DNA
CRP: C-reactive protein
csbDNA: Cell surface-bound DNA
DAS: Disease Activity Score
DAS28: 28-joint Disease Activity Score
dNTP: Deoxynucleotide triphosphate
EDTA: Ethylenediaminetetraacetic acid
ELISA: Enzyme-linked immunosorbent assay
EULAR: European League Against Rheumatism
HS: Healthy subjects
IgG: Immunoglobulin G
LINE-1: Long interspersed nuclear element 1
LR: Likelihood ratio
m-cirDNA: Cell-free mitochondrial circulating DNA
m-csbDNA: Mitochondrial cell surface-bound DNA
MP: Microparticle
MTX: Methotrexate
n-cirDNA: Cell-free nuclear circulating DNA
n-csbDNA: Nuclear cell surface-bound DNA
nDNA: Nuclear DNA
NET: Neutrophil extracellular trap
PCA: Principal component analysis
PCR: Polymerase chain reaction
PMN: Polymorphonuclear neutrophil
RA: Rheumatoid arthritis
RF: Rheumatoid factor
SLE: Systemic lupus erythematosus
TAMRA: 5,6-Carboxytetramethylrhodamine
