# Supplementary material for: Circulating DNA in rheumatoid arthritis: pathological changes and association with clinically used serological markers
Source: Arthritis Res Ther. 2017 May 2;19:85. doi: 10.1186/s13075-017-1295-z (PMC5414163; doi:10.1186/s13075-017-1295-z)
Supplement: Supplementary file 1 — Correlation of n-cirDNA, m-cirDNA, n-csbDNA, m-csbDNA, ACPA, CRP, and RF between each other and with age of patients with rheumatoid arthritis. (DOC 220 kb) [file 13075_2017_1295_MOESM1_ESM.doc]

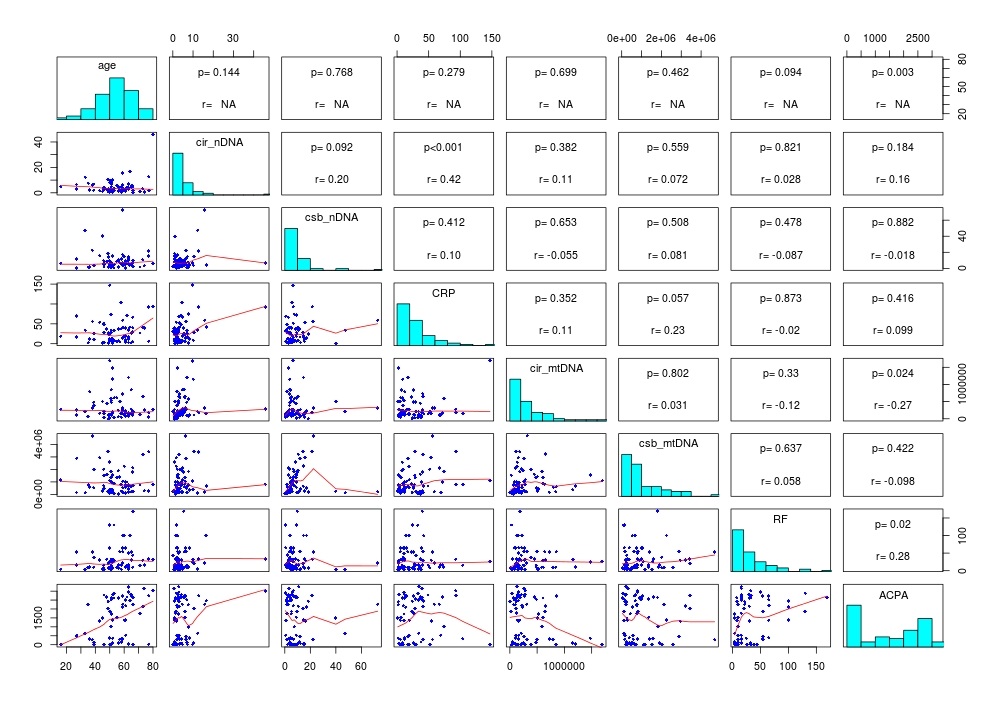
Figure S1.

**Correlation** of n-cirDNA, m-cirDNA, n-csbDNA, m-csbDNA, ACPA, CRP, RF between each other and with age of **patients with rheumatoid arthritis**. Correlation coefficients (r) and p values (p) from the Spearman rank-order test are displayed.
