# Supplementary material for: Circulating DNA in rheumatoid arthritis: pathological changes and association with clinically used serological markers
Source: Arthritis Res Ther. 2017 May 2;19:85. doi: 10.1186/s13075-017-1295-z (PMC5414163; doi:10.1186/s13075-017-1295-z)
Supplement: Supplementary file 2 — Correlation of n-cirDNA, m-cirDNA, n-csbDNA, m-csbDNA, ACPA, CRP, and RF between each other and with age of healthy subjects. (DOC 219 kb) [file 13075_2017_1295_MOESM2_ESM.doc]

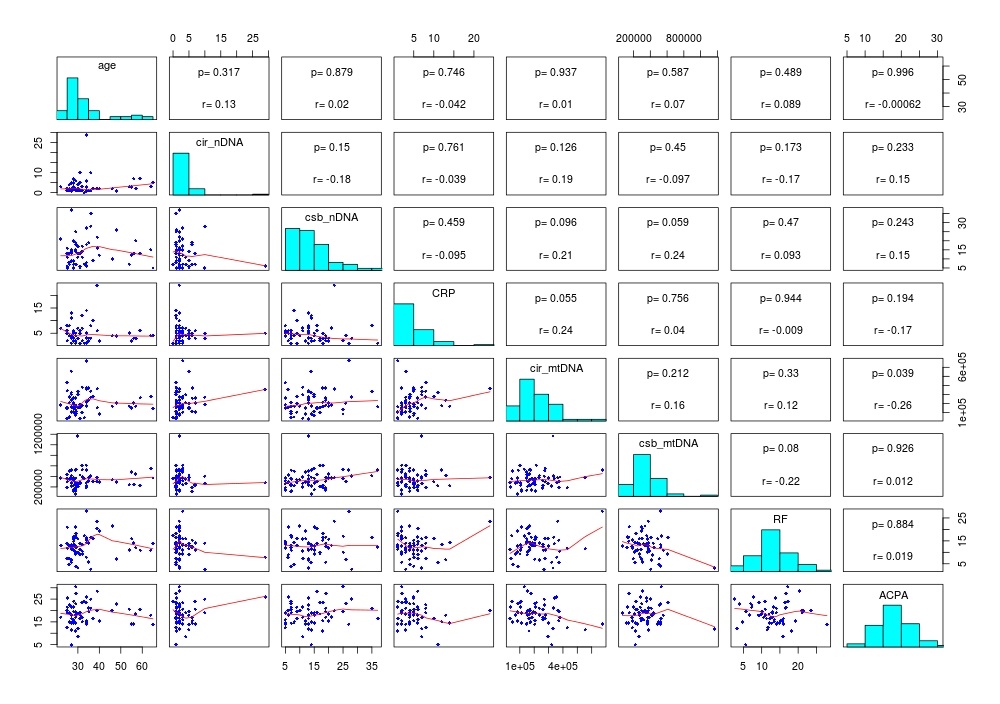


Figure S2

**Correlation** of n-cirDNA, m-cirDNA, n-csbDNA, m-csbDNA, ACPA, CRP, RF between each other and with age of **healthy subjects**. Correlation coefficients (r) and p values (p) from the Spearman rank-order test are displayed.
