# Supplementary material for: Circulating DNA in rheumatoid arthritis: pathological changes and association with clinically used serological markers
Source: Arthritis Res Ther. 2017 May 2;19:85. doi: 10.1186/s13075-017-1295-z (PMC5414163; doi:10.1186/s13075-017-1295-z)
Supplement: Supplementary file 3 — Comparison of cir-nDNA, m-cirDNA, n-csbDNA, and m-csbDNA concentration in the blood from patients with recent-onset, established, and end-stage rheumatoid arthritis. (DOC 31 kb) [file 13075_2017_1295_MOESM3_ESM.doc]

**Table S1.** Comparison of cir-nDNA, m-cirDNA, n-csbDNA, m-csbDNA concentration in the blood from patients with recent-onset, established and end-stage of rheumatoid arthritis

| Groups/variables | N-cirDNA^a^ | N-csbDNA^a^ | M-cirDNA^b^ | M-csbDNA^b^ | ACPA^c^ | RF^c^ | CRP^c^ |
| --- | --- | --- | --- | --- | --- | --- | --- |
| Patients with recent-onset RA | 8.8  (2.8 — 20.4) | 22.4  (7.2 — 62.4) | 0.15 x 10^6^  (0.07 — 0.52) x 10^6^ | 0.57 x 10^6^  (0.03 — 1.16) x 10^6^ | 1375  (12 —  1725) | 4.0  (0.4 — 8.1) | 11.3  (4.6 - 37) |
| Patients with established RA | 12.0  (0.1 — 16.6) | 21.2  (0.4 — 40.0) | 0.2 x 10^6^  (0.03 — 1.7) x 10^6^ | 0.77 x 10^6^  (0.07 — 3.42) x 10^6^ | 1621  (4 —  3152) | 23.1  (4.0 — 130.3) | 19.1  (1.4 — 147.1) |
| Patients with end-stage RA | 12.0  (0.68 — 45.7) | 26.4  (0.7 — 72.7) | 0.18 x 10^6^  (0.003 — 0.79) x 10^6^ | 0.6 x 10^6^  (0.1 — 4.68) x 10^6^ | 1758  (22 —  3254) | 16.1  (4.0 — 169) | 27.4  (2.9 —  139.1) |
| Subgroups: recent-onset  vs established | p>0.05 | p>0.05 | p>0.05 | p>0.05 | p>0.05 | p<0.01 | p>0.05 |
| Subgroups: established vs end-stage | p>0.05 | p>0.05 | p>0.05 | p>0.05 | p>0.05 | p>0.05 | p>0.05 |
| Subgroups:  recent-onset vs end-stage | p>0.05 | p>0.05 | p>0.05 | p>0.05 | p=0.11 | p<0.01 | p<0.05 |

^a^ n-cirDNA and n-csbDNA concentrations, ng/ml of blood;

^b^ m-cirDNA and m-csbDNA concentrations, copies/ml of blood;

^c^RF, ACPA and CRP concentrations, U/ml of blood plasma; median values are represented and range in the brackets;
