# Supplementary material for: Circulating DNA in rheumatoid arthritis: pathological changes and association with clinically used serological markers
Source: Arthritis Res Ther. 2017 May 2;19:85. doi: 10.1186/s13075-017-1295-z (PMC5414163; doi:10.1186/s13075-017-1295-z)
Supplement: Supplementary file 4 — cir-nDNA, m-cirDNA, n-csbDNA, and m-csbDNA concentration in the blood from patients treated with methotrexate plus etoricoxib in comparison with rituximab plus methotrexate. (DOC 49 kb) [file 13075_2017_1295_MOESM4_ESM.doc]

Table S2**.** Concentrations of circulating nuclear DNA and mitochondrial DNA in the blood from RA patients treated with methotrexate/etoricoxib or with rituximab/ methotrexate

| Groups for comparison | Cir-nDNA^c^ | Csb-nDNA^c^ | Cir-mtDNA^d^ | Csb-mtDNA^d^ |
| --- | --- | --- | --- | --- |
| RA patients group 1^a^ | 12.0  (0.4 – 183.2) | 24.0  (1.8 – 290.8) | 0.38 x 10^6^  (0.006 – 3.38) x 10^6^ | 1.44 x 10^6^  (0.06- 9.36) x 10^6^ |
| RA patients group 2^b^ | 12.8  (2.4 – 43.2) | 19.2  (6.8 – 160.4) | 0.44 x 10^6^  (0.012 – 2.98) x  10^6^ | 0.93 x 10^6^  (0.06- 9.36) x 10^6^ |
| Group 1 vs group 2 | p=0.60 | p=0.64 | p=0.71 | p=0.15 |

^a^ – RA patients group 1 (n=74) (see Materials and Methods)

^b^ – RA patients group 2 (n=14) (see Materials and Methods)

^c^ n-cirDNA and n-csbDNA concentrations, ng/ml of blood;

^d^ m-cirDNA and m-csbDNA concentrations, copies/ml of blood;
