# Supplementary material for: Circulating DNA in rheumatoid arthritis: pathological changes and association with clinically used serological markers
Source: Arthritis Res Ther. 2017 May 2;19:85. doi: 10.1186/s13075-017-1295-z (PMC5414163; doi:10.1186/s13075-017-1295-z)
Supplement: Supplementary file 5 — Variable importance evaluated using random forests classification algorithm. (DOC 28 kb) [file 13075_2017_1295_MOESM5_ESM.doc]

**Table S3. V**ariable importance evaluated using Random Forest classification algorithm

| Variable | Mean Decrease Accuracy |
| --- | --- |
| N-cirDNA | 23.5 |
| N-csbDNA | 33.6 |
| M-cirDNA | 26.3 |
| M-csbDNA | 78.8 |
| C-reactive protein | 64.1 |
| ACPA | 132.1 |
| Rheumatoid factor | 56.7 |
